# Knowledge about tooth avulsion and its management among dental assistants in Riyadh, Saudi Arabia

**DOI:** 10.1186/1472-6831-14-46

**Published:** 2014-05-06

**Authors:** Hassan Suliman Halawany, Yousra Hussain AlJazairy, Nawaf Sulaiman Alhussainan, Nassr AlMaflehi, Vimal Jacob, Nimmi Biju Abraham

**Affiliations:** 1 Department of Periodontics and Community Dentistry, College of Dentistry, King Saud University, Riyadh, Kingdom of Saudi Arabia; 2 Dental Caries Research Chair, College of Dentistry, King Saud University, Riyadh, Kingdom of Saudi Arabia; 3 Department of Restorative Dentistry, College of Dentistry, King Saud University, Riyadh, Kingdom of Saudi Arabia; 4 Dental Health Department, Prince Sultan Military Medical City, Riyadh, Kingdom of Saudi Arabia

**Keywords:** Avulsion, Knowledge, Dental assistant

## Abstract

**Background:**

Studies evaluating dental assistants’ knowledge about tooth avulsion and its management are rare. The purpose of this study was to evaluate the level of knowledge about tooth avulsion and its management among dental assistants in Riyadh, Saudi Arabia and to assess its relationship with their educational background.

**Methods:**

A convenience sampling methodology was employed for sample selection. Over a period of four months starting in February, 2013, 691 pretested 17-item questionnaires were distributed. A total of 498 questionnaires were returned for an overall response rate of 72.1%. Six questions were related to knowledge about permanent tooth avulsion and one question was related to knowledge about primary tooth avulsion. Correct answers to these questions were assigned one point each, and based on this scoring system, an overall knowledge score was calculated. An analysis of covariance was used to test the association between the level of knowledge (total score) and the educational qualifications of the respondents (dental degree and others). A P-value of 0.05 was considered the threshold for statistical significance.

**Results:**

The majority of the respondents (n = 387; 77.7%) were non-Saudis (377 were from the Philippines), and 79.1% (n = 306) of the Filipinos had a dental degree. The question about recommendations for an avulsed tooth that is dirty elicited the highest number of correct responses (n = 444; 89.2%), whereas the question about the best storage media elicited the lowest number of correct responses (n = 192; 38.6%). The overall mean score for knowledge about tooth avulsion was 6.27 ± 1.74. The mean knowledge score among the respondents with a dental degree was 6.63 ± 1.37, whereas that among the respondents with other qualifications was 5.71 ± 2.08.

**Conclusions:**

The educational qualifications of the surveyed dental assistants were strongly correlated with the level of knowledge about tooth avulsion and its management.

## Background

Dental trauma is the most common of all facial injuries, and avulsion occurs in 1-16% of all dental injuries [1,2]. The procedures performed at the time of tooth avulsion and the extra-alveolar time immediately after the incident determines the prognosis of the tooth [1]. In cases in which the above mentioned factors are unfavorable, pulp necrosis and degeneration of the periodontal ligament (PDL) cells can occur, leading to inflammatory root resorption and eventual tooth loss [2]. The maintenance of PDL cell vitality favors successful tooth replantation [3]. However, although it is the treatment of choice [1], replantation cannot always be performed immediately.

In 2012, the International Association of Dental Traumatology (IADT) developed and published a consensus statement on the guidelines for managing avulsed permanent teeth, which describes current evidence and practice based on the literature and expert opinion. The primary objective of these guidelines is to outline an approach for the instantaneous care of avulsed permanent teeth and to aid dentists and other healthcare professionals in decision-making in the event of permanent teeth avulsion. Although the IADT does not guarantee that firm adherence to the guidelines will produce favorable outcomes, applying this protocol maximizes the chances of a favorable outcome [1]. Oral healthcare professionals are expected to be familiar with the management of avulsed teeth so that they can provide the best possible treatment for their patients and participate in the education of their communities. Several studies have attempted to assess the knowledge of various populations (including dentists, physicians, schoolteachers, parents and athletic coaches) regarding the emergency management of avulsed teeth [4-9]. Most of these studies have highlighted the need for better communication between dental professionals and the community and for the effective implementation of educational campaigns. A Saudi Arabian study conducted in 1997 reported a higher prevalence of dental trauma among 5- to 6-year-old boys and 12- to 14-year-old boys than that reported by the United Kingdom Children’s Dental Health Survey in the same age groups [10]. Another study reported that the prevalence of dental trauma to the maxillary incisors among 12- to 15-year-old Saudi schoolgirls was 31.4%, which is considered high [11]. Furthermore, a study conducted by Al-Obaida [12] concluded that the majority of Saudi teachers in Riyadh city did not know how to manage a child who had sustained a dental injury.

Dental assistants are an integral part of the auxiliary dental team, and they can be consulted directly or by telephone during the initial emergency management of avulsed teeth. Studies evaluating the knowledge of dental assistants concerning tooth avulsion and its management have been rare. Although a study conducted by Cohenca et al. [4] evaluated the knowledge of oral health professionals regarding the treatment of avulsed teeth, the majority of their sample consisted of general dentists; the number of dental assistants included in this study was relatively low. Consequently, the aim of our study was to evaluate the level of knowledge about tooth avulsion and its management among dental assistants in Riyadh, Saudi Arabia and to assess its relationship with their educational background.

## Methods

The present study was registered with and approved by the College of Dentistry Research Center (CDRC; registration number FR 0035) and the study was undertaken with the understanding and written consent of each subject according to the ethical principles of the World Medical Association Declaration [13]. A 17-item English-language questionnaire, which was a modified version of the questionnaire used by Cohenca et al. for their study of specialized oral health professionals, general dentists and dental assistants [4], was developed for this study of dental assistants working in Saudi Arabia. The questionnaire was pretested in a group of ten randomly selected Saudi and non-Saudi dental assistants to identify any comprehension problems, and modifications were made accordingly. The questionnaire consisted of seven demographic items and ten items regarding dental trauma and its management. The survey was conducted with dental assistants working in various public and private hospitals, clinics, polyclinics and dental schools in Riyadh, Saudi Arabia.

A convenience sampling methodology was employed for sample selection. Over a period of four months starting in February 2013, 691 questionnaires were distributed. Each prospective participant was approached individually by two of the co-investigators, who assessed his or her willingness to participate voluntarily in the study by completing the anonymous questionnaire. The participants were not required to divulge their names or any other identifying information. The people who were willing to participate were given the questionnaire, which was completed and returned immediately.

According to 2013 statistics from the Saudi Commission for Health Specialties, the total number of registered dental assistants working in the Kingdom of Saudi Arabia is 3,790, and the total number in Riyadh is 1,364. More specifically, there are 409 Saudi dental assistants and 955 non-Saudi dental assistants (of whom 936 are from the Philippines) working in Riyadh [14]. A considerable proportion of the Filipino dental assistants have a dental degree (Doctor of Dental Medicine from the Philippines). Considering the fact that the majority of non-Saudi dental assistants working in Riyadh are from the Philippines, the educational qualifications of the sample were categorized as dental degree or non-dental degree (all other qualifications). Nationality was categorized as Saudi or non-Saudi, current employment was categorized as private or public, years of experience was categorized as less than or equal to ten years or more than ten years of experience and working hours per day were categorized as less than or equal to eight hours or more than eight hours for the statistical analyses.

The data obtained from the completed questionnaires were entered into and analyzed using the Statistical Package for the Social Sciences software, version 20.0 for Windows (IBM; SPSS Inc., Chicago, IL, USA). Descriptive statistics and χ^2^ tests were used to summarize and analyze the data, respectively. Six questions were related to knowledge about permanent tooth avulsion and one question was related to knowledge about primary tooth avulsion. Questions 3 through 9 were used to assess the respondents’ level of knowledge about tooth avulsion using a scoring system. Apart from the first seven questions on demographics, questions 1, 2 and 10 were not suitable for scoring (refer to the Additional file 1). Thus, six questions (question numbers 3, 4, 5, 6, 7 and 9) were assigned one point each for correct answers and zero points for wrong answers. For question number 8, one point was assigned for each of the four suitable types of storage media (refer to the italics in the Additional file 1 for the point distributions). The maximum possible score was ten points. An analysis of covariance was used to test the association between the level of knowledge about avulsion and its management (total score) and the educational qualifications of the respondents (dental degree or non-dental degree). Binary logistic regression was used to assess the significance of each background variable (educational qualifications, age, sex, nationality, years of experience, current employment, working hours per day and previous advice or education on tooth avulsion) in explaining the likelihood of answering each knowledge-based question correctly. A P-value of 0.05 was considered the threshold for statistical significance.

## Results

Of the 691 questionnaires distributed, 498 were returned with data suitable for analysis, for an overall response rate of 72.1%. The majority of the respondents (n = 387; 77.7%) were non-Saudis, and 79.1% (n = 306) of them had a dental degree. Of the 387 non-Saudis, 377 were from the Philippines, four were from Sudan, and one each was from Yemen, Syria, Pakistan, India, the United Kingdom and the United States of America. The demographic characteristics of the study population are shown in Table 1.

**Table 1:** Demographic characteristics of the study population

**Variables**		**Total N = 498 n (%)**
Sex	Male	81 (16.3)
	Female	417 (83.7)
Age (years old)^1^	34.19 ± 8.70 (Mean ± SD)	
	≤30	198 (41.9)
	31-40	154 (32.6)
	41-50	102 (21.6)
	51-60	18 (3.8)
Educational qualifications^§^	Bachelor of Dental Assisting	51 (10.2)
	Diploma in Dental Assisting	67 (13.5)
	Dental degree	306 (61.4)
	BSc Nursing	53 (10.6)
	Others	21 (4.2)
Nationality	Saudi	111 (22.3)
	Non-Saudi	387 (77.7)
Current employment	Public	349 (70.1)
	Private	149 (29.9)
Years of experience^2^	<5 years	204 (41.8)
	5-10 years	135 (27.7)
	11-15 years	79 (16.2)
	16-20 years	45 (9.2)
	>20 years	25 (5.1)
Working hours per day	≤8 hours	181 (36.6)
	>8 hours	313 (63.4)

^§^Highest educational qualification obtained; ^1^26 missing values; ^2^10 missing values.

The majority of the participants had received advice or education on tooth avulsion (n = 372; 75%) and had seen cases in which a permanent tooth was avulsed (n = 312; 63%). The percentage distributions of the responses to the general knowledge questions about avulsion and its management are shown in Table 2. The majority of the subjects (n = 325; 65.3%) correctly responded that an avulsed permanent tooth should not be replaced in its socket under all circumstances. Most of the respondents (n = 354; 72.5%) reported correctly that an avulsed primary tooth should not be replanted, and approximately 88% (n = 436) reported that they would seek immediate dental treatment for the avulsed tooth.

**Table 2:** Percentage distributions of the responses to questions about avulsion by the educational qualifications

**General knowledge about avulsion and its management**		**Dental degree; n (%)**	**Non-dental degree; n (%)**	**N = 498; n (%)**
Should an avulsed permanent tooth be replaced n its socket?	Yes, in all cases	70 (22.9)	54 (28.1)	124 (24.9)
	Not in all cases	222 (72.5)	103 (53.6)	325 (65.3)
	Never	7 (2.3)	10 (5.2)	17 (3.4)
	I don’t know	7 (2.3)	25 (13.0)	32 (6.4)
Do you think that an avulsed primary tooth should be replanted?^1^	Yes	71 (23.6)	63 (33.7)	134 (27.5)
	No	230 (76.4)	124 (66.3)	354 (72.5)
How urgently do you think one should seek dental treatment for an avulsed tooth?^2^	Immediately	284 (93.1)	152 (79.6)	436 (87.9)
	Within a few hours	11 (3.6)	11 (5.8)	22 (4.4)
	Before 24 hours has elapsed	7 (2.3)	13 (6.8)	20 (4.0)
	I don’t know	3 (1.0)	15 (7.9)	18 (3.6)

^1^10 missing values; ^2^2 missing values.

The percentage distributions of the responses to the specialized knowledge questions about avulsion and its management are shown in Table 3. The majority of the respondents (n = 444; 89.5%) recommended rinsing the tooth gently under running tap water for a few seconds without scrubbing it if the avulsed tooth was dirty. Furthermore, most of the evaluated dental assistants (n = 427; 86.4%) reported that they would hold the crown only while washing the avulsed tooth. Fresh milk (n = 192; 38.6%), followed by saline solution (n = 116; 23.3%) and the patient’s mouth/saliva (n = 111; 22.3%) were reported to be the best storage media for an avulsed tooth. However, 98 subjects (19.7%) incorrectly reported that tap water was an appropriate storage medium for an avulsed tooth.

**Table 3:** P**ercentage distributions of the responses to questions about management of avulsion by the educational qualifications**

**Specialized questions about avulsion and its management**		**Dental degree; n (%)**	**Non-dental degree; n (%)**	**N = 498; n (%)**
If you were informed of an avulsion and that the tooth is dirty, what would you recommend?^1^	Wipe the tooth with tissue paper	3 (1.0)	16 (8.3)	19 (3.8)
	Clean the tooth with a toothbrush	2 (0.7)	7 (3.6)	9 (1.8)
	Rinse gently for a few seconds under running tap water without scrubbing it	298 (98.0)	146 (76.0)	444 (89.5)
	I don’t know	1 (0.3)	23 (12.0)	24 (4.8)
How would you hold the tooth while washing it?^2^	Hold the crown only	283 (93.4)	144 (75.4)	427 (86.4)
	Hold the root only	0 (0.0)	5 (2.6)	5 (1.0)
	Hold the crown or root	16 (5.3)	23 (12.0)	39 (7.9)
	I don’t know	4 (1.3)	19 (9.9)	23 (4.7)
Which storage media are suitable for storing an avulsed tooth?^§^	Tap water	62 (20.3)	36 (18.8)	98 (19.7)
	Patient’s mouth/saliva	146 (47.7)	72 (37.5)	218 (43.8)
	Tissue paper/cotton	13 (4.2)	11 (5.7)	24 (4.8)
	Fresh milk	216 (70.6)	137 (71.4)	353 (70.9)
	Saline solution	194 (63.4)	95 (49.5)	289 (58.0)
	Antiseptic solution	20 (6.5)	15 (7.8)	35 (7.0)
	Hank’s balanced salt solution	50 (16.3)	34 (17.7)	84 (16.9)
	I don’t know	1 (0.3)	10 (5.2)	11 (2.2)
Which is the best storage medium?	Fresh milk	102 (33.3)	90 (46.9)	192 (38.6)
	Patient’s mouth/saliva	84 (27.5)	27 (14.1)	111 (22.3)
	Saline solution	74 (24.2)	42 (21.9)	116 (23.3)
	Hank’s balanced salt solution	27 (8.8)	15 (7.8)	42 (8.4)
	Others	15 (4.8)	11 (5.8)	26 (5.2)
	I don’t know	4 (1.3)	7 (3.6)	11 (2.2)

^§^More than one answer was possible; therefore, the results are expressed as numbers and percentages of respondents answering each option; ^1^2 missing values; ^2^4 missing values.

A statistically significant association was found between question 1 and educational qualifications (χ^2^ = 23.034; P = 0.000). A total of 252 respondents with a dental degree (82.4%) and 120 (63.2%) participants with other qualifications (non-dental degree) reported having received previous advice or education on tooth avulsion, whereas 54 (17.6%) subjects with a dental degree and 70 (36.8%) with other qualifications reported that they did not receive such advice. Furthermore, a statistically significant association was found between question 2 and educational qualifications (χ^2^ = 15.795; P = 0.000). A total of 213 (69.8%) evaluated dental assistants with a dental degree and 99 (52.1%) respondents with other qualifications had seen cases in which a permanent tooth was avulsed, whereas 92 (30.2%) subjects with a dental degree and 91 (47.9%) respondents with other qualifications had not.

The overall mean score for knowledge about tooth avulsion was 6.27 ± 1.74. An analysis of covariance was performed to assess the differences in the level of knowledge about avulsion (total score) between the respondents with a dental degree and those with other qualifications, controlling for variables such as age, sex, experience, employment and working hours per day. A highly significant difference (P = 0.000) was found in the total knowledge score between the participants with a dental degree and those with other qualifications (Table 4). The mean knowledge score among the respondents with a dental degree was 6.63 ± 1.37, whereas the mean knowledge score among the subjects with other qualifications was 5.71 ± 2.08.

**Table 4:** Analysis of covariance – test of between-subjects effects

**Source**	**Type III sum of squares**	**F**	**P value**	**Observed power**
Corrected	96.285^a^	5.632	0.000	0.997^a^
Intercept	271.520	95.293	0.000	1.000
Age	0.549	0.193	0.661	0.072
Sex	1.602	0.562	0.454	0.116
Experience	0.294	0.103	0.748	0.062
Employment	1.166	0.409	0.523	0.098
Working hours	0.310	0.109	0.742	0.063
Education	59.308	20.815	0.000 vhs	0.995
Error	1290.748			
Total	19481.000			
Corrected total	1387.033			

^a^R squared = 0.069 (Adjusted R squared = 0.057).

Computed using α = 0.05.

Dependent variable: Total score.

Between-subjects factor: Education (Dental degree and non-dental degree).

vhs: very highly significant.

A significant association was found between question 10 and years of experience (χ^2^ = 4.660; P = 0.031); a higher percentage of the respondents (n = 127; 37.8%) with less than ten years of experience reported that they were not adequately informed about traumatic dental injuries, including avulsion. A significant association was also found between questions 10 and 1 (χ^2^ = 53.002; P = 0.000); a higher percentage of the participants (n = 273; 85.8%) who received previous advice or education about tooth avulsion reported that they were adequately informed about traumatic dental injuries, including avulsion.

The logistic regression analysis for each knowledge-based question revealed that knowledge about tooth avulsion differed with regard to the background characteristics of the participants and their previous education concerning tooth avulsion (question 1), as shown in Table 5. The numbers in parentheses below are odds ratios.

**Table 5:** Knowledge about tooth avulsion: Odds ratio based on maximum-likelihood estimates using logistic regression

	**Edu (1)**	**Age**	**Sex (1)**	**Nation (1)**	**Exp (1)**	**Employ (1)**	**Work hrs (1)**	**Question 1 (1)**
Replantation of avulsed permanent tooth (Question 3)	1.29 (0.70-2.37)	1.04 (1.00-1.08)	1.13 (0.64-1.97)	**0.44** (0.21-0.89)	1.44 (0.76-2.73)	0.86 (0.50-1.47)	1.17 (0.76-1.80)	1.01 (0.62-1.64)
Replantation of avulsed primary tooth (Question 4)	**1.98** (1.07-3.69)	1.02 (0.98-1.06)	1.13 (0.62-2.04)	1.77 (0.84-3.74)	0.95 (0.49-1.84)	1.66 (0.93-2.99)	1.09 (0.69-1.72)	1.01 (0.60-1.69)
Urgency in seeking treatment (Question 5)	1.58 (0.63-3.96)	1.00 (0.94-1.06)	1.67 (0.68-4.09)	0.52 (0.19-1.41)	1.13 (0.41-3.13)	0.94 (0.40-2.18)	**0.50** (0.27-0.95)	**3.34** (1.74-6.41)
Recommendations for an avulsed tooth that is dirty (Question 6)	**10.92** (3.15-37.84)	1.04 (0.97-1.12)	1.03 (0.42-2.51)	0.64 (0.23-1.79)	2.25 (0.56-9.14)	1.99 (0.72-5.51)	1.11 (0.55-2.24)	**3.13** (1.53-6.40)
Handling avulsed tooth (Question 7)	**2.98** (1.28-6.96)	1.03 (0.96-1.09)	2.27 (0.97-5.33)	0.63 (0.24-1.62)	0.78 (0.24-2.58)	0.79 (0.35-1.76)	1.77 (0.91-3.44)	**4.17** (2.22-7.82)
Suitable storage medium (Patient’s saliva)	**1.90** (1.03-3.51)	0.97 (0.93-1.00)	1.04 (0.61-1.79)	1.25 (0.60-2.59)	0.83 (0.46-1.47)	0.94 (0.56-1.57)	0.96 (0.64-1.45)	**2.91** (1.76-4.82)
Suitable storage medium (Fresh milk)	1.23 (0.66-2.28)	**0.96** (0.92-0.99)	1.12 (0.63-1.99)	1.41 (0.63-3.12)	1.00 (0.53-1.88)	0.81 (0.46-1.44)	0.93 (0.59-1.47)	**4.00** (2.43-6.58)
Suitable storage medium (Saline)	1.04 (0.58-1.88)	1.01 (0.98-1.05)	1.10 (0.64-1.88)	0.51 (0.25-1.04)	0.74 (0.41-1.32)	1.06 (0.64-1.78)	1.02 (0.67-1.53)	**0.50** (0.31-0.82)
Suitable storage medium (HBSS)	1.00 (0.48-2.08)	0.99 (0.95-1.04)	**2.50** (1.37-4.59)	0.90 (0.35-2.29)	1.20 (0.52-2.75)	**2.32** (1.22-4.42)	0.78 (0.44-1.38)	1.33 (0.71-2.51)
Best storage medium (Fresh milk)	0.71 (0.39-1.29)	1.02 (0.98-1.05)	0.90 (0.52-1.57)	1.69 (0.83-3.46)	**1.98** (1.09-3.59)	0.99 (0.59-1.66)	1.16 (0.76-1.75)	**2.40** (1.44-3.99)

Confidence interval (lower, upper) in parentheses. The variables denoting the numbers in bold were statistically significant (P < 0.05).

Education: (1) – Dental degree, (0) – Non-dental degree; Sex: (1) – Male, (0) – Female; Nationality: (1) – Saudi, (0) – Non-Saudi; Experience: (1) - ≤10 years, (0) - >10 years; Working hours per day: (1) - ≤8 hours, (0) >8 hours; Question 1 (Have you ever received advice or education on tooth avulsion?): (1) – Yes, (0) – No.

The likelihood of answering most of the knowledge-based questions correctly was significantly greater among the respondents who had received previous advice or education on tooth avulsion. They were more than 3 times more likely to know that dental treatment for an avulsed tooth should be sought immediately (3.34) and to recommend the correct management of an avulsed tooth if it was dirty (3.13). They were 4 times more likely to describe the correct handling of an avulsed tooth before replantation (4.17) and also to identify fresh milk as a suitable storage medium (4.00). Conversely, they were less likely to identify saline as a suitable storage medium compared with those who had not received previous advice or education on tooth avulsion (0.50).

The educational qualifications of the dental assistants also played a significant role in determining the maximum likelihood estimates. The participants with a dental degree were nearly twice as likely to report that an avulsed primary tooth should not be replanted (1.98) and to describe the correct handling of an avulsed tooth before replantation (2.98). In addition, they were nearly 11 times more likely to recommend the correct management of an avulsed tooth that was dirty (10.92) compared with the dental assistants with other qualifications. The patient’s saliva was almost twice more likely to be selected as a suitable storage medium by the respondents with a dental degree than were those with other qualifications (1.98).

The likelihood of selecting Hank’s balanced salt solution (HBSS) as a suitable storage medium was more than twice as likely to be reported by males (2.50) and those working in private institutions (2.32) compared with females and those working in public institutions, respectively. Fresh milk was nearly twice as likely to be reported as the best storage medium by those with less than ten years of work experience compared with those with more than ten years of work experience (1.98).

## Discussion

This cross-sectional study was conducted to evaluate the level of knowledge about tooth avulsion and its management among dental assistants in Riyadh, Saudi Arabia and to assess its relationship with their educational background. There was a very significant difference in the total knowledge scores between the respondents with a dental degree and the respondents with other qualifications. The majority of our respondents were non-Saudis, mostly from the Philippines, with a dental degree; these individuals are considered licensed dentists in the Philippines.

Approximately 70% of our respondents with a dental degree had never encountered a case of tooth avulsion, which is greater than the reported percentages among dentists (60%) in the United States [4]; among other professionals, such as teachers, in Istanbul, Turkey (35.8%) and in Porto, Portugal (23%) [15]; and among physicians (24%) in India [16]. A study conducted by Al-Obaida [12] among Saudi primary schoolteachers reported that only 22.7% of the surveyed teachers had seen at least one case of avulsion in their teaching careers. Furthermore, approximately 52% of our respondents with a non-dental degree had never come across a case of tooth avulsion, which is greater than the rate reported among physicians and teachers in the above-mentioned studies and among school nurses (42%) in a study conducted by Baginska and Wilczynska-Borawska [17]. However, it should be noted that the question asked in the study conducted by Baginska and Wilczynska-Borawska [17] was specific to tooth avulsion experiences at work, which was not the case in our study. Tooth avulsion can occur anywhere, so our finding that a higher percentage of our respondents had encountered a case of tooth avulsion should be interpreted with caution.

The majority of the respondents in this study (65.3%) reported that an avulsed permanent tooth should not be replanted in all cases, which is in accordance with the results of the studies conducted by Cohenca et al. [4] and Zhao and Gong [18], in which 53.3% and 72.8% of the participating dentists, respectively, had the same opinion. The current IADT guidelines [1] provide examples of individual situations in which replantation is not indicated, including cases of severe caries or periodontal disease, cases in which the patient is not cooperative and cases in which the patient has certain severe medical conditions, such as immunosuppression or a severe cardiac condition. Regarding replantation of an avulsed primary tooth, the majority of the respondents in this study (72.5%) reported that they would not replant an avulsed primary tooth. This response, which concurs with the current guidelines of IADT [1], was given by 83.3% of the participants in the study conducted by Cohenca et al. [4], 83% of the participants in the study conducted by Stokes et al. [9] and 87.1% of the participants in the study conducted by Zhao and Gong [18]. The majority of the respondents in this study (87.9%) also reported that dental treatment should be sought immediately for an avulsed tooth, which is in agreement with the results of Cohenca et al. [4], who reported that 81.3% of their respondents recognized the urgency of seeking professional care as soon as the avulsion occurs (within 30 minutes). Furthermore, 88.5% of the participants in the study conducted by Zhao and Gong [18] reported that the critical period is 30 minutes and that an avulsed tooth should be replanted within this interval. In a study conducted by Loh et al. [19] among dental therapists in Singapore, all of the respondents (100%) indicated that immediate action was needed for traumatized teeth, including avulsed teeth.

According to the current IADT guidelines, an avulsed tooth that is dirty should be washed briefly (for 10 seconds) under cold running water [1]. The majority of our respondents (89.5%) reported that they would rinse an avulsed tooth that is dirty gently under running tap water for a few seconds without scrubbing it, which is greater than the reported rate among dentists and primary schoolteachers [4,12]. In response to the question about how the respondents would hold the avulsed tooth while washing it, the majority of our respondents (86.4%) reported that they would hold the crown only. The study conducted by Baginska and Wilczynska-Borawska [17] reported that 94% of surveyed school nurses would hold an avulsed tooth by the crown.

The current IADT guidelines mention HBSS, saline, milk and saliva as examples of osmolality-balanced media that are appropriate for storing avulsed teeth [1]. Consequently, one point was assigned to the respondents for reporting each of these media as suitable for storing avulsed teeth (question 8) in the calculation of the knowledge score. Fresh milk was suggested as a suitable storage medium by 70.9% of our respondents, followed by saline (58%) and the patient’s mouth/saliva (43.8%). Approximately 39% of our respondents reported that fresh milk was the best storage medium, followed by saline (23.3%) and the patient’s mouth/saliva (22.3%). The majority of the participants (53.6%) in the study conducted by Cohenca et al. [4] identified fresh milk as the recommended transport medium for an avulsed tooth from the site of injury to the dental office. However, our results are not in accordance with those of the studies conducted by Baginska and Wilczynska-Borawska among school nurses [17] and dentists [20], in which the majority of the respondents (82% and 88.7%, respectively) reported that saline was the recommended transport medium. Because fresh milk is the most commonly available osmolality-balanced storage medium, one point was assigned to the respondents in our study who identified fresh milk as the best storage medium (question 9). The study conducted by Loh et al. [19] reported that although 97.6% of the dental therapists participating in their study recognized that an avulsed tooth should be stored in a suitable medium for transportation, 54.8% did not know the correct medium to use. Approximately 20% of our respondents wrongly reported that tap water was a suitable storage medium. In a study conducted by Abu-Dawoud et al. [21], gauze, ice, warm water, tap water, cold water and disinfectant were wrongly reported by 90%, 90%, 76.6%, 53.3%, 43.3% and 33.3% of physicians, respectively, and tap water and warm water were wrongly reported by 76.6% and 43.3% of dentists, respectively, as some of the methods and media that can be used to store an avulsed tooth.

It is not surprising that a significantly greater number of our respondents with a dental degree had received previous advice or education on tooth avulsion, had seen cases in which a permanent tooth had been avulsed and had higher mean knowledge scores compared with the respondents with other qualifications. This may be due to the fact that the respondents with a dental degree are considered licensed dentists in their home country (the Philippines). Furthermore, it was more likely that they would have encountered a case of an avulsed tooth before starting their careers as dental assistants compared with the respondents with other qualifications. However, the question asked in our study did not specifically ask whether they had seen a case of avulsion before or after starting their careers as dental assistants. Although the mean knowledge score among the respondents with a non-dental degree (5.71 ± 2.08) was significantly lower than the score among those with a dental degree (6.63 ± 1.37) in this study, the overall mean knowledge score was 6.27 ± 1.74. This finding is comparable to the results of the study conducted by Baginska and Wiczynska-Borawska [17], who reported a mean score of 5.16 ± 1.69 among school nurses who had never been trained in dental trauma management and a mean score of 6.6 ± 1.3 among those who had attended a lecture on first aid for victims of dental trauma two years before the study was conducted. The educational qualifications of the respondents (i.e., whether they had a dental degree) and receiving previous advice or education on tooth avulsion were significant variables in explaining the likelihood of answering a majority of the knowledge-based questions correctly in our logistic regression model.

Although the cross-sectional design, a convenience sampling methodology instead of random sampling and the lack of a control group could be considered limitations of this study, the sample size and the response rate indicate that our study included a representative sample of dental assistants working in Riyadh, Saudi Arabia. The lack of differentiation between ‘encountering’ tooth avulsion and actually treating/managing tooth avulsion (question 2) may be considered a drawback. The questionnaire was developed to survey Saudi and non-Saudi dental assistants working in Riyadh; these assistants are not legally entitled to treat patients. Consequently, question 2 was modified to suit the study population. Furthermore, the lack of calibration of the survey instrument may also be considered a potential limitation of this study.

## Conclusions

Highly qualified and well-trained dental assistants are an important asset for practicing dentists and dental assistants with greater knowledge about critical issues, such as the management of tooth avulsion, will be an added advantage. Although educational qualifications and previous advice or education about tooth avulsion were strongly correlated with the level of knowledge about tooth avulsion and its management, there was no significant association with our respondents’ experience as dental assistants. Consequently, it may be desirable for Saudi dentists to consider educational qualifications and participation in continuing educational programs on critical issues such as the management of tooth avulsion when hiring dental assistants. Regular continuing education programs on dental trauma and its management will certainly improve dental assistants’ knowledge and ability to manage avulsion injuries. Based on the results of this study, future studies including general dental practitioners and non-dental clinical providers such as physicians and nurses as control groups will provide us with a broader perspective on the level of knowledge about tooth avulsion.

## Competing interests

The authors declare that they have no competing interests.

## Authors’ contributions

HSH conceptualized the study and was involved with the study design, YHA was involved with the study design and data collection, NSA was involved with the study design and data collection, NAM was involved with the study design and data analysis, VJ was involved with the study design and writing up of the manuscript, NBA was involved with the study design, data compilation and writing up of the manuscript. All authors read and approved the final manuscript.

## Pre-publication history

The pre-publication history for this paper can be accessed here:

http://www.biomedcentral.com/1472-6831/14/46/prepub

## Supplementary Material

Additional file 1 Questionnaire. Click here for file
