# Supplementary material for: Knowledge about tooth avulsion and its management among dental assistants in Riyadh, Saudi Arabia
Source: BMC Oral Health. 2014 May 6;14:46. doi: 10.1186/1472-6831-14-46 (PMC4107998; doi:10.1186/1472-6831-14-46)
Supplement: Additional file 1 — Questionnaire. [file 1472-6831-14-46-S1.docx]

**Appendix**

**Demographic details:**

Age: …………………………. Gender: Male Female

Nationality: ………………………………………………….

Educational qualifications: ………………………………………………………………….

Years of experience: <5 years 5 – 10 years 11 – 15 years

16 – 20 years >20 years

Current employment: Private Public

Working hours per day: ≤8 hours >8 hours

**Knowledge about tooth avulsion and its management:**

1. Have you ever received advice or education on tooth avulsion?
   1. Yes
   2. No
2. Have you ever seen a case where a permanent tooth has been avulsed?
   1. Yes
   2. No
3. Should an avulsed **permanent tooth** be replaced in its socket?
4. Yes, in all cases
5. Not in all cases
6. Never
7. I don’t know

*(one point for choosing b, zero points for choosing any other option)*

1. Do you think that an avulsed **primary tooth** should be replanted?
   1. Yes
   2. No

*(one point for choosing b, zero points for choosing a)*

1. How urgent do you think it is to seek dental treatment for an avulsed tooth?
   1. Immediately
   2. Within a few hours
   3. Before 24 hours has elapsed
   4. I don’t know

*(one point for choosing a, zero points for choosing any other option)*

1. If you found the avulsed tooth and it is dirty, what would you recommend?
   1. Wipe the tooth with a tissue paper
   2. Clean the tooth with a toothbrush
   3. Rinse gently for few seconds without scrubbing it under running tap water
   4. I don’t know

*(one point for choosing c, zero points for choosing any other option)*

1. How do you hold the tooth while washing it?
   1. Hold the crown only
   2. Hold the root only
   3. Crown or root
   4. I don’t know

*(one point for choosing a, zero points for choosing any other option)*

1. Which of the following storage media **are** suitable for storing an avulsed tooth? (choose all possible alternatives)
   1. In tap water
   2. In the patient’s mouth/ saliva
   3. Wrapped in tissue paper/ cotton
   4. In fresh milk
   5. In saline solution
   6. In antiseptic solution
   7. Hank’s balanced salt solution
   8. I don’t know

*(one point each for choosing b, d, e, or g and zero points for choosing any other options)*

1. From the above options, which is **the best** storage medium for an avulsed tooth before seeking care, according to you?

*(one point for choosing d, zero points for choosing any other options)*

1. Do you feel adequately informed about traumatic dental injuries, including avulsion?
   1. Yes
   2. No
